# Antimicrobial activity of novel nanostructured Cu-SiO_2_ coatings prepared by chemical vapour deposition against hospital related pathogens

**DOI:** 10.1186/2191-0855-3-53

**Published:** 2013-09-05

**Authors:** Sajnu Varghese, Souad O ElFakhri, David W Sheel, Paul Sheel, Frederick J Eric Bolton, Howard A Foster

**Affiliations:** 1 Centre for Parasitology and Disease Research, School of Environment and Life Sciences, University of Salford, Salford M5 4WT, UK; 2 Materials and Physics Research Centre, University of Salford, Salford M5 4WT, UK; 3 CVD Technologies Ltd., Manchester, UK; 4 Health Protection Agency, PO Box 209, Clinical Sciences Building, Manchester Royal Infirmary, Oxford Road, Manchester M13 9WZ, UK

**Keywords:** Antimicrobial, Chemical vapour deposition, Copper, Disinfection surface, Hospital pathogens

## Abstract

There is increasing recognition that the healthcare environment acts as an important reservoir for transmission of healthcare acquired infections (HCAI). One method of reducing environmental contamination would be use of antimicrobial materials. The antimicrobial activity of thin silica-copper films prepared by chemical vapour deposition was evaluated against standard strains of bacteria used for disinfectant testing and bacteria of current interest in HCAI. The structure of the coatings was determined using Scanning Electron Microscopy and their hardness and adhesion to the substrate determined. Antimicrobial activity was tested using a method based on BS ISO 22196:2007. The coatings had a pale green-brown colour and had a similar hardness to steel. SEM showed nano-structured aggregates of Cu within a silica matrix. A log10 reduction in viability of >5 could be obtained within 4 h for the disinfectant test strains and within 6 h for producing *Acinetobacter baumannii*, *Klebsiella pneumoniae* and *Stenotrophomonas maltophilia*. Activity against the other hospital isolates was slower but still gave log10 reduction factors of >5 for extended spectrum β-lactamase producing *Escherichia coli* and >3 for vancomycin resistant *Enterococcus faecium*, methicillin resistant *Staphylococcus aureus* and *Pseudomonas aeruginosa* within 24 h. The results demonstrate the importance of testing antimicrobial materials destined for healthcare use against isolates of current interest in hospitals as well as standard test strains. The coatings used here can also be applied to substrates such as metals and ceramics and have potential applications where reduction of microbial environmental contamination is desirable.

## Introduction

It is now accepted that environmental contamination plays a key role in the transmission of infectious diseases in the healthcare setting (Hota, 2004; Boyce, 2007; Bartley and Olmsted, 2008; Dancer, 2009; Weber *et al*., 2010; Otter *et al*., 2011). There is a higher risk of acquiring a HCAI if the previous room occupant had such an infection (Shaughnessy *et al*., 2011). Pathogens can survive on surfaces for prolonged periods of time depending on the organism and environmental conditions (Kramer *et al*., 2006; Neely and Maley, 2000; Wagenvoort *et al*., 2011) and can be transmitted to hands from the environment (Bhalla *et al*., 2004). Enhanced environmental cleaning has been shown to reduce rates of infection (Dancer *et al*., 2009; Kochar *et al*., 2009; Carling *et al*., 2010; Kleypas *et al*. 2011). However, the environment rapidly becomes recontaminated following disinfection (Hardy *et al*., 2007).

One possible approach to controlling environmental contamination has been the reintroduction of copper (Cu) into hospitals (Sasahara *et al*., 2007; Casey *et al*., 2010; Mikolay *et al*., 2010; Espirito Santo *et al*., 2011; Schmidt *et al*., 2012) and other healthcare settings (Marais *et al*. 2010). Cu is widely used for its antimicrobial properties (Borkow and Gabbay, 2009; Grass *et al*., 2011). Cu and Cu alloy surfaces have been shown to kill a variety of pathogens including *Salmonella enterica* and *Campylobacter jejeuni* (Faundez *et al*., 2004), *Listeria monocytogenes* ( Wilks *et al.,*^2006^), methicillin resistant *Staphylococcus aureus* (MRSA; Noyce *et al*., 2006a; Gould *et al*., 2009; Michels *et al*., 2009; Weaver *et al*., 2010), *Escherichia coli* O157 (Wilks *et al*., 2005; Noyce *et al*., 2006b), *Mycobacterium tuberculosis* (Mehtar *et al*., 2008), *Clostridium difficile* (Wheeldon *et al*., 2008; Weaver *et al*., 2008), *Pseudomonas aeruginosa* (., Gould *et al*^2009^) and enterococci (Gould *et al*., 2009; Warnes and Keevil, 2011). A crossover study compared conventional surfaces for a toilet seat, tap handles and a ward entrance door push plate with Cu containing items (Casey *et al*., 2010). The study showed reduced (>90%) bacterial counts on the items containing Cu and no indicator pathogens (e.g. VRE) were isolated during the 5 week study period on the copper whereas they were detected on control surfaces. A further longer study in an intensive care unit showed a sustained reduction of 83% in microbial surface counts on six commonly touched Cu containing surfaces and numbers on control surfaces were also reduced (Schmidt *et al*., 2012). This was accompanied by a reduction in the rates of infection on the unit (Salgado *et al*., 2013). Recolonisation of Cu surfaces following cleaning has been shown to be delayed compared to control surfaces (Mikolay *et al*., 2010). However, Cu surfaces may become conditioned allowing colonisation following cleaning (Airey and Verran, 2007) possibly by retention and survival of organisms in surface scratches (Verran *et al*., 2010).

Sol–gel methods can be used to producing durable Cu containing glasses but these have not been investigated for their antimicrobial properties (Perez-Robles *et al*., 1999; Tohidi *et al*., 2010). Coatings with antimicrobial activity containing Cu nanoparticles have been prepared by surface coating with a powder mixture followed by heat treatment to fuse the particles into a glass-like coating (Esteban-Tejeda *et al*., 2012). In the present study chemical vapour deposition (CVD) was used to produce thin film coatings. In this process a reactive gas mixture containing coating precursors is introduced into a coating region and a source of energy e.g. heat applied to initiate (or accelerate) decomposition of the precursor and growth of the coating on the target substrate (Choy, 2003). Atmospheric Pressure CVD (APCVD) has been widely used e.g. for production of self-cleaning coatings for glass which generally have excellent hardness and durability. We have previously reported the use of CVD to produce antimicrobial SiO_2_ (silicon dioxide) coatings containing silver (Ag; Cook *et al*., 2011; Varghese *et al*., 2013). In this report we describe the activity of similar coatings but containing Cu instead of Ag as the antimicrobial component. The activity of these Cu-SiO_2_ (copper-silicon dioxide) coatings against hospital related pathogens that are of current interest was investigated.

## Materials and methods

### Microorganisms and growth conditions

*Escherichia coli* ATCC 8739, and *Staphylococcus aureus* ATCC6538 were obtained from the American Type Culture Collection. *Pseudomonas aeruginosa* 10421 was obtained from the National Collection of Industrial and Marine Bacteria, Aberdeen U.K. and strain AOH1 was a wild isolate recovered from the river Tame downstream from Greenfield Wastewater Treatment Works. Extended spectrum β-lactamase (ESBL)^+^*Acinetobacter baumannii*, KPC^+^ (carbapenemase) *Klebsiella pneumoniae*, ESBL^+^*Escherichia coli*, EMRSA15, two recent isolates of MRSA, MRSA 1599 and MRSA 1665, MRSA NCTC10492, *Stenotrophomonas maltophilia*, and vancomycin resistant *Enterococcus faecium* (VRE) were obtained from the Health Protection Agency, Manchester, U.K. and sub-cultured onto Nutrient Agar (NA, Oxoid, Basingstoke, UK) and incubated at 37°C for 24 h. Cultures were resuspended in Nutrient Broth (NB, Oxoid) and kept on Microban® beads (TCS Ltd Merseyside, UK) at −70°C. Prior to use, one bead was sub-cultured onto NA and incubated at 37°C for 24 h.

### Production of coatings

Cu-SiO_2_ coatings were deposited on 1 mm borosilicate glass (Dow Corning) using flame assisted chemical vapour deposition (FACVD). The FACVD system was of in-house design and construction and consisted of a brass burner head above a translational stage and a precursor delivery system of ultrasonic nebuliser, bubbler and mass flow controllers (Cook *et al*., 2011). Tetraethylorthosilicate was carried to the burner head using a nitrogen flow rate of 0.5 lmin^-1^ from a heated and stirred bubbler (75°C ± 3°C, stirred at 120 rpm). An aqueous solution of copper sulphate (0.25 M) was used as the copper precursor and simultaneously delivered to the burner head by ultrasonically nebulising the aqueous solution prior to carriage by nitrogen at 0.6 lmin^-1^. The number of passes under the burner head was 6 equating to a residence time in the flame of approximately 12 sec and gave a film approx 25 nm thick.

In the later stages of the study a new coating head was used which was capable of coating 10 cm wide substrates. Copper content of the films was varied by changing the concentration of the precursor and the flow rate to the coating head. The different conditions for the coatings are shown in Table 1.

**Table 1:** Coating conditions used in this study and their physical characteristics

**Coating number**	**Cu(NO**_**3**_**)**_**2 **_**precursor concentration M**	**Flow rate to burner head l min**^**-1**^	**Mohs hardness**	**Transmission %**^**#**^
1	0.25	0.6	5.6	90.7
2	0.25	2*	3.6	87.9

*Second coater head. ^#^Control glass had 91.5% transmission.

### Characterisation of coatings

To assess the hardness of the deposited coatings, films were scratch tested using a constant load scratch hardness tester. A diamond tipped scribe was moved through 50 mm over the surface with a 100 g load. The mean width of the resulting scratch over 6 points was then measured under 200× optical magnification and compared to similar data from materials of known Mohs hardness ( aluminium, steel, copper, glass and quartz) and Mohs hardness values of the deposited films were calculated. Results are the means of three determinations.

Adhesion of the coating to the substrate was determined by Scotch tape testing. The coating was cross hatched every 5 mm with a diamond scribe, the adhesive tape was then applied and pressed firmly to ensure consistent contact with the coating. On removal the tape was observed visually and then under a microscope to determine if the integrity of the film had been maintained.

### Appearance of the films

Transmission of the coatings in visible light was measured using an Aquilla NKD7000 spectrometer using plane polarized light source and transmission averaged over 400-700 nm and measured at a 30° angle.

Surface morphology was investigated using Scanning Electron Microscopy (SEM; Philips XL30) with samples sputter coated with a 2–3 nm layer of Pt/Pd to provide a conductive surface.

### Testing for antimicrobial activity

Antimicrobial activity was tested based on BS ISO 22196:2007 (Anon. 2007) except that glass covers were used rather than plastic, the test was done at 20-25°C rather than 35°C (see discussion) and samples were tested after different times rather than just after 24 h as specified in the test. Twenty mm square samples of coated and control glass were sterilized by placing in 90% methanol for 20 min. The squares were transferred to a sterile Petri dish and left for at least 1 h to allow the methanol to evaporate. Colonies were resuspended in a 1:500 dilution of NB and adjusted to OD 0.01-0.02 at 600 nm in a spectrophotometer (Camspec, M330, Cambridge, UK) to give approx. 2×10^7^ colony forming units (cfu) cm^-3^. Fifty μl was inoculated on to each test sample and covered with an 18 mm square of 1 mm borosilicate glass to ensure close contact between the culture and the film. The samples were placed in 50 mm diameter Petri dishes containing moistened filter paper to prevent drying out of the suspensions. Plain borosilicate glass was used for controls. Samples were removed after 0, 1, 2, 4, 6 and 24 h and immersed in 20 cm^3^ of sterile Tryptone Soy broth (Oxoid) together with the cover glass and vortexed for 60 sec to resuspend the bacteria. A viability count was performed by dilution and plating on NA in triplicate and incubation at 37°C for up to 48 h. TSB had previously been shown to inactivate copper released from the surfaces at up to 1 mM by incubation of cultures in TSB supplemented with CuSO_4_.5H_2_O (data not shown).

In order to determine the effects of protein on antimicrobial activity, bovine serum albumen (BSA: Sigma-Aldrich, Poole, Dorset, UK) was added to the 1:500 NB used for production of the test culture suspensions before inoculation onto test and control surfaces at a final concentration of 10 gl^-1^.

### Statistical analysis

Where possible each experiment was done in triplicate and means and standard deviations calculated using Microsoft Excel. Survival curves were plotted as the means with standard deviations as error bars. In order to allow plotting survival curves on a logarithmic scale, because zero cannot be plotted on a logarithmic scale, one was added to each mean viable count. In some cases error bars were obscured by the graph symbols and in others only upper error bars were plotted.

## Results

**Figure 1:**
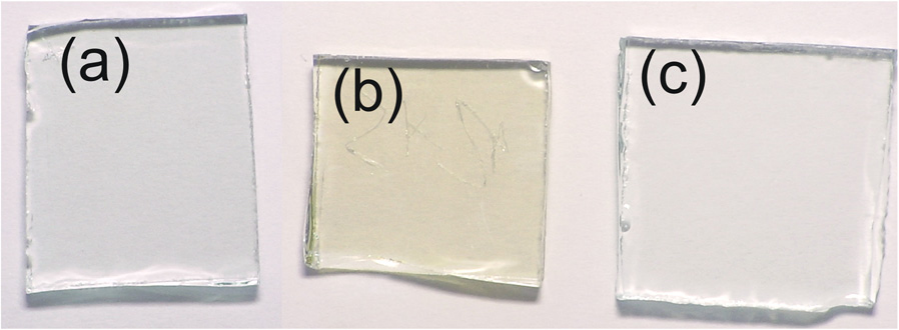
**Visual appearance of coated glass. (a)** Coating 1, **(b)** coating 2, **(c)** control glass

**Figure 2:**
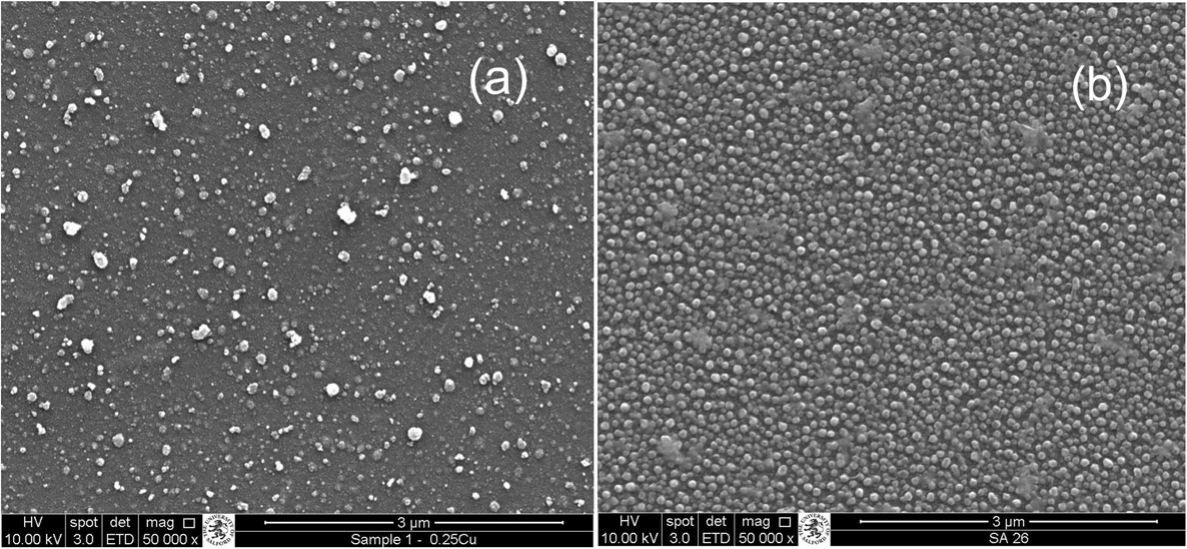
**SEM analysis of CuO-SiO**_**2 **_**coated glass. (a)** coating 1 **(b)** Coating 2.

The films had a pale brown-green tinge which was darker with the higher Cu content (Figure 1). Transmission in the visible range was 88-91% compared to 91.5% for the control glass (Table 1). The Cu incorporation reduced the transmission as expected especially with higher concentrations of Cu. SEM showed an amorphous background with evenly distributed aggregates which increased in size and number with higher concentrations of Cu (Figure 2).

The Mohs hardness of films were 5.6 for coating 1 and 3.6 for coating 2 giving a hardness comparison to stainless steel for coating 1 (stainless steel = 5.5). For all films the coating remained intact in the Scotch tape test showing a good adhesion to the substrates. Coating 1 was used in most subsequent experiments, coating 2 was only used to study the effects of protein on antimicrobial activity.

**Figure 3:**
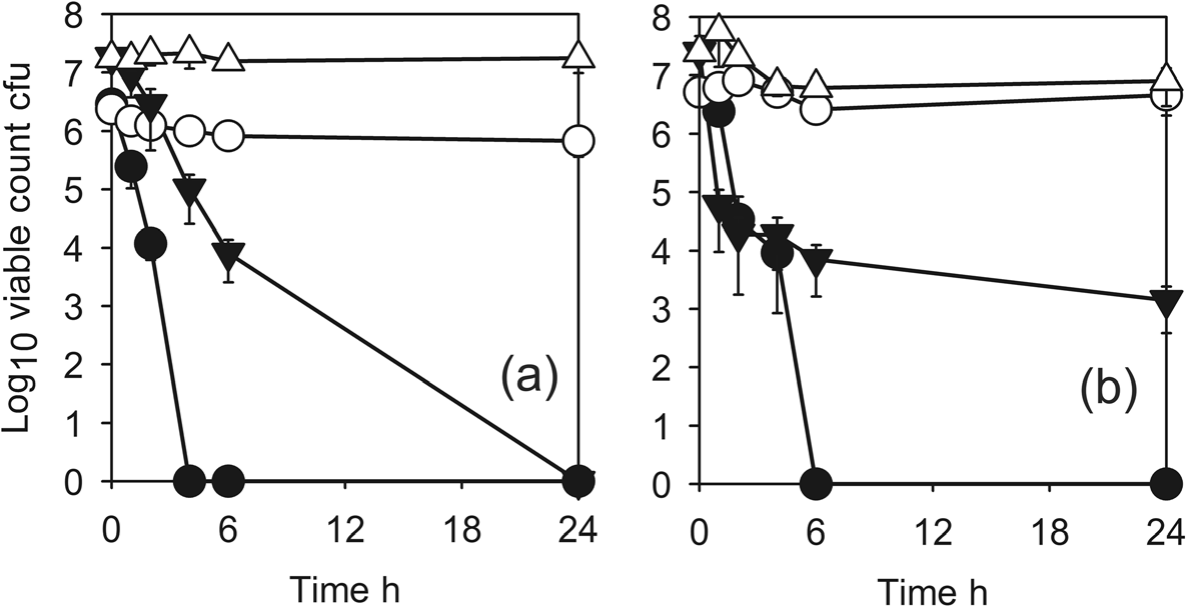
**Antimicrobial activity of Cu-SiO**_**2 **_**coating against *Escherichia coli *and *Pseudomonas aeruginosa*(d)*. *(a)** ● *Escherichia coli* ATCC8739 (disinfectant test strain) test; ◯, ATCC8739 control; ▼, ESBL *E. coli* test; ▽, ESBL *E. coli* control ◯. **(b)** ●; *Pseudomonas aeruginosa* 10421 (disinfectant test strain) test; ◯, *P. aeruginosa* 10421 control, ▼, *P. aeruginosa* AOH1 (wild isolate) test; ▽, *P. aeruginosa* AOH1 control. Coating 1.

The antimicrobial activity of coating 1 against the standard test strain of *E. coli* (ATCC8739) is shown in Figure 3a. There was a log10 reduction factor of >5 after 4 h. The ESBL producing *E. coli* was more resistant and it took 24 h to obtain a similar reduction. The standard strain of *P. aeruginosa* was reduced by a log10 reduction factor of 5 after 6 h but the wild isolate AOH1 was only reduced by a factor of 3.5.

**Figure 4:**
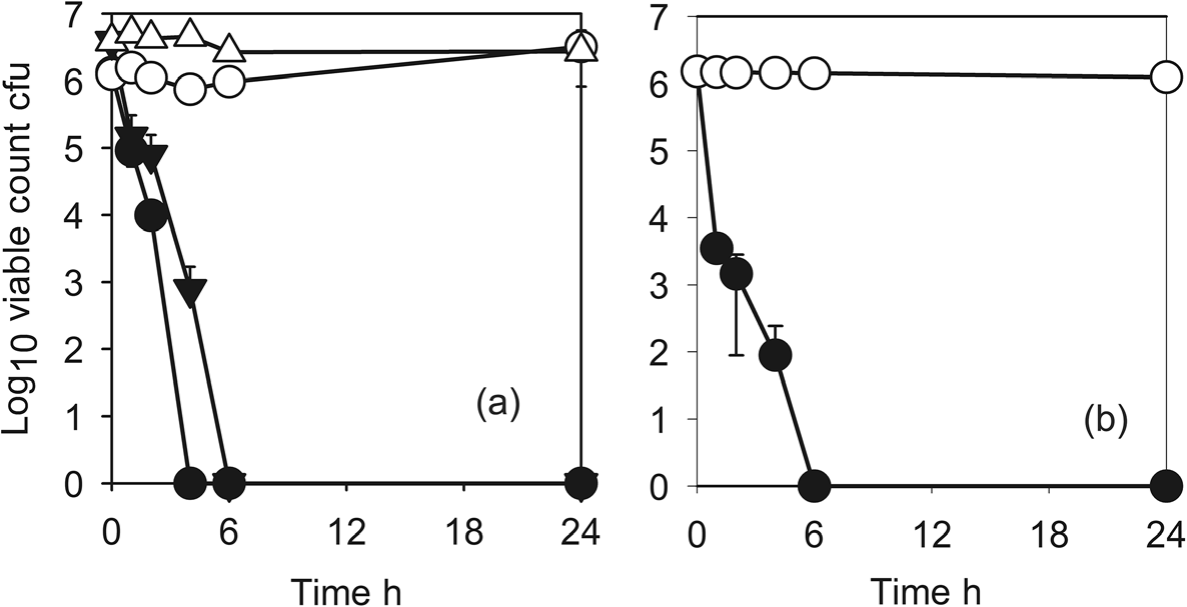
**Antimicrobial activity of CuO-SiO**_**2 **_**coating against Gram-negative pathogens. (a)** ●, ESBL^*+*^*Acinetobacter baumannii test*; ◯, *A.baumannii* control; ▼*Stenotrophomonas maltophilia* test; ▽, *S.maltophilia* control. **(b)**●, KPC^+^*Klebsiella pneumoniae* test; ◯, *K. pneumoniae* control. Coating 1.

**Figure 5:**
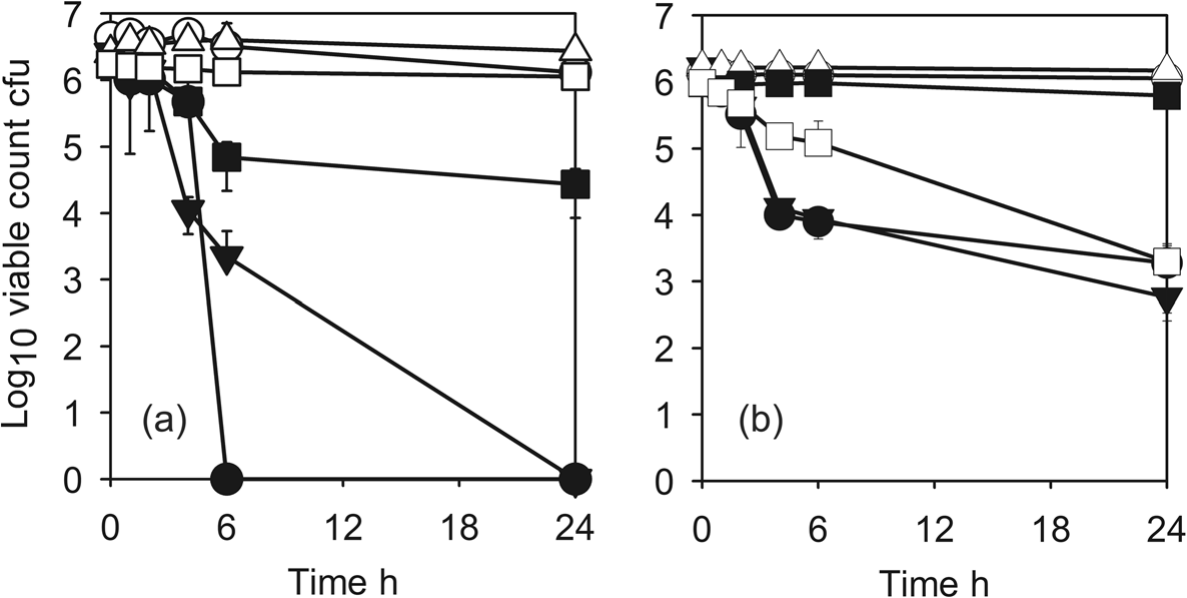
**Antimicrobial activity of Cu-SiO**_**2 **_**coating against *Staphylococcus aureus *and vancomycin resistant *Enterococcus faecium*. (a)** ●, *Staphylococcus aureus* ATCC 9538 (methicillin sensitive disinfectant test strain) test; ◯, *Staphylococcus aureus* ATCC 9538 control; ■, EMRSA15 test; □, EMRSA15 control; ▼*Staphylococcus aureus* NCTC 12493 (reference MRSA) test; ▽ *Staphylococcus aureus* NCTC 12493 control. **(b)** □ Vancomycin resistant *Enterococcus faecium* test; ■, Vancomycin resistant *E. faecium* control; ◯, MRSA1599 test; ●, MRSA1599 control;▼ MRSA 1669 test; ▽, MRSA1669 control. Coating 1.

The activity against the Gram-negative clinical isolates is shown in Figure 4. ESBL producing *A. baumannii* and *S. maltophilia* (Figure 4a) and *K. pneumoniae* (Figure 4b) all had log10 reduction factors of >5 in 4–6 h. The activity of the coatings against Gram-positive organisms is shown in Figure 5. The disinfectant test strain of *S.aureus* (ATCC9538) was reduced by a log10 reduction factor of >5 after 6 h (Figure 5a). MRSA strains were more resistant with a log10 reduction factors of >5 for the type strain NCTC10492 after 24 h (Figure 5a) and approx. 3 after 24 h for EMRSA15 and the two recent clinical isolates MRSA1595 and 1669 (Figures 5a, b). The vancomycin resistant *E. faecium* gave a similar reduction (Figure 5b).

**Figure 6:**
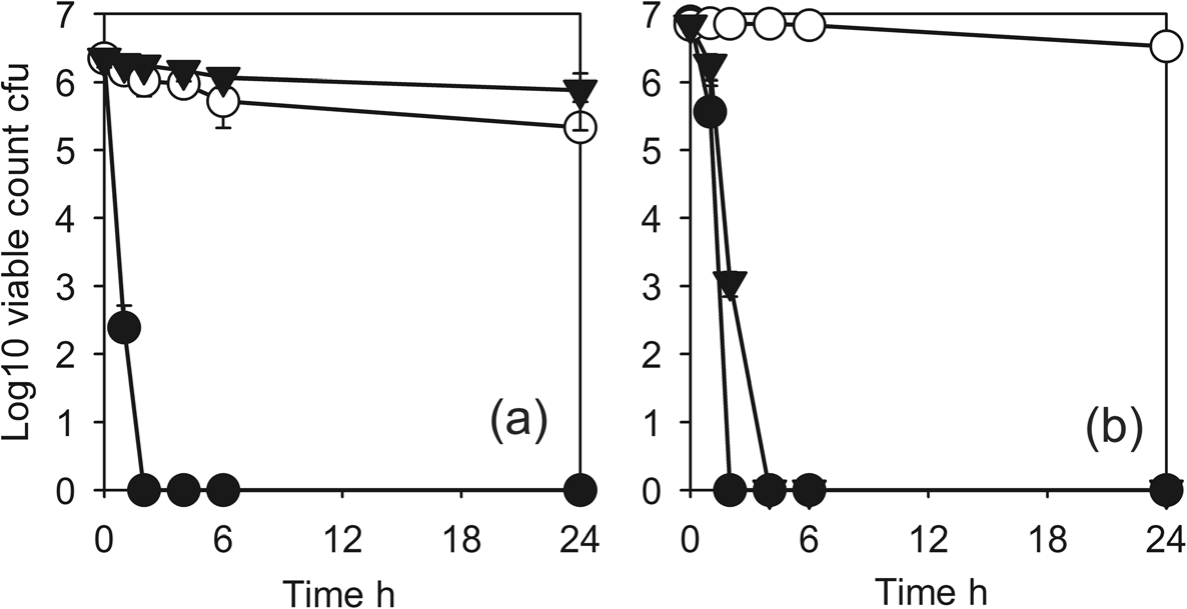
**Effects of protein on antimicrobial activity of Cu-SiO**_**2 **_**coating against *Escherichia coli *ATCC 10536. (a)** Coating 1 ●, no protein; ▼, 10gl^-1^ bovine serum albumen; ◯, control. **(b)** coating 2 ●, No protein; ▼, 10gl^-1^ bovine serum albumen; ◯, control.

The effects of addition of protein on the antimicrobial activity against *E. coli* ATCC9739 are shown in Figure 6. With coating 1 the activity was completely inhibited and there was no killing even after 24 h. With coating 2, which had a higher Cu content, the activity was slowed but there was a log10 reduction factor of >5 after 6 h.

## Discussion

The structure of the films under SEM suggests that there are islands of Cu which extend above the surface of the film. This is consistent with our previous observations of Ag containing CVD coatings where a clear island structure was observed for Ag only films and in Ag-SiO_2_ composite films we identified embedded silver nano-particles which demonstrated a degree of crystallinity (Cook *et al*., 2011). The scotch tape test and scratch testing showed that the coatings were well attached to the substrate and had abrasion resistance equivalent to steel. This is important in the resistance to wear during the expected lifetime of the products (10–15 y). The benefit of using CVD is that good properties are achievable with a relatively simple process. Capital costs for equipment are also relatively low. These points are a significant advantage versus, for example, sputtering processes. Although we did consider some other ceramics (e.g. tungsten oxide, molybdenum oxide) we chose to use silica because we had done a lot of previous work on this matrix by FACVD (Cook *et al*., 2011, Varghese *et al,*^2013^) and silica and silica precursors are compatible with FACVD.

The results show that organisms such as ESBL producing *K. pneumoniae* and *A. baumannii* were killed relatively quickly on the Cu-SiO_2_ coating. ESBL *E. coli* was more resistant than the standard test strain but was still effectively killed within 24 h. The MRSA strains were much more resistant than the Gram-negative bacteria and were also more resistant than the standard test strain of *S. aureus* or even than a type strain of MRSA used for testing methicillin resistance. A similar result was obtained for MRSA with titanium dioxide-copper coatings (Foster *et al*., 2012) and may reflect increased resistance of MRSA to killing by copper. It should be noted that the numbers or organisms used in the BS ISO method are much higher than have been reported in environmental contamination during outbreaks of MRSA (2.5 × 10^5^ vs 500 cfu cm^-2^; Otter *et al*., 2011) although in the latter case the organisms may not have been evenly distributed. The activity detected would still reduce the MRSA to less than 1 cfu cm^-2^ as suggested/recommended by Dancer (2011) within 6 h provided that the activity determined here was maintained in real use situations. The other Gram-positive bacterium tested was a vancomycin resistant *E. faecium* strain (VRE). This proved as resistant to the CuO-SiO_2_ coatings as the MRSA although the reduction was equivalent to a 99.8% reduction after 24 h. Preliminary tests with extended incubation showed a log 10 reduction of >5 after 48 h for MRSA and VRE. VRE have been shown to be capable of persisting in the environment for months and even years (Neely and Maley, 2000; Wagenvoort *et al*., 2011). The use of antimicrobial coatings in a healthcare setting should help to reduce this persistence.

Preliminary studies showed that Cu did elute from the Cu-SiO_2_ coated surfaces (as Cu ions) and reached 20–70 μM in the thin liquid films formed during the antimicrobial testing (determined by ICPMS, M. Abohtera and H.A. Foster unpublished). Relatively few studies report the bactericidal concentrations for Cu against bacteria and the value varies depending on medium composition and time of exposure. The minimum inhibitory concentration (MIC) for copper sulphate for several species of bacteria isolated from food animals was 20 mM for the majority of strains of *E. coli* and from 2–12 mM for *S. aureus* and *Staphylococcus hyicus*. *Enterococcus* spp. had a bimodal distribution with MIC from 2–24 mM (Aarestrup and Hasman 2004). Concentrations of eluted Cu^2+^ from the CVD coated surfaces are therefore much lower than MIC but there may be locally higher concentrations near to the surface of the coating. Alternatively, contact between the bacterium and the copper islands may allow diffusion of Cu^2+^ directly into the cell wall/membrane. Various mechanisms have been suggested for the antimicrobial activity of Cu including membrane damage, inhibition of respiration, protein inactivation and damage to DNA (Borkow and Gabbay, 2009; Grass *et al*., 2011). The mechanism may be different for bacteria with different cell-wall structure with membrane damage predominant in Gram-negative bacteria and inhibition of respiration and DNA damage in Gram-positive bacteria (Warnes and Keevil, 2011, 2012). This may in part explain the differences in rates of killing seen here.

Interfering agents e.g. protein may be present in real use situations and even the low concentrations used here reduced the activity. Inhibition by amounts of protein that may be present in e.g. serum or food may be much higher. These surfaces will need to be cleaned to remove any such contamination but this is true of copper surfaces which can become conditioned in actual use (Airey and Verran, 2007). Increasing the amount of copper in the coating reduces the inhibitory effect of protein but the hardness of the coating is also reduced. The nature of the coatings used for *in situ* applications will need to be a compromise between durability and activity.

We used a modification of the BS ISO 22196 method to allow determination of the rates of killing and we used room temperature to reflect activity at normal temperatures. Preliminary results suggested that activity was increased at higher temperatures giving an inflated impression of activity compared to room temperature. This method does not reflect the natural contamination that may occur *in situ* and there is an urgent need for standardised methods that reflect this as has been suggested by previous authors (O’Gorman and Humphreys 2012; Grass *et al*., 2011). However, the test does give information on the relative antimicrobial activity of the films against different organisms. A true test will be to determine the performance of the coatings *in situ* and also long term tests of durability and activity e.g. when subjected to washing and disinfection will need to be confirmed and these are currently under investigation.

The results suggest that application of the coatings to surfaces in the Healthcare setting may provide a useful background antimicrobial activity which functions continually and which may help reduce the inevitable recontamination which occurs following even the most penetrating disinfection treatments e.g. hydrogen peroxide vapour fogging (Hardy *et al*., 2007). Environmental contamination has been shown to be important in transmission of a number of organisms including *A. baumannii* (Aygün *et al*., 2002) and VRE (Martinez *et al*., 2003; Hayden *et al*., 2008). The coatings may therefore have a role in reduction of transmission of such organisms in the healthcare setting, particularly as they can be applied to different materials e.g. glass, ceramic tiles and metals. Indeed the surfaces that could be coated are only limited by the temperature reached during the coating process (approx 150°C) substrates would have to withstand this temperature. This will provide a range of materials for different uses which will be complementary to the use of copper and copper alloys and should contribute to the overall reduction in microbial contamination of the hospital environment with an associated reduction in transmission of infections. Used together with antimicrobial paints, fabrics, plastics and floor-coverings they may help to make the dream expressed by Bennett (2008) of a “self-disinfecting ward” a closer reality. These surfaces will not replace the normal cleaning and disinfection regimes but they will provide additional protection between such treatments. They may also find applications in other situations where control of microbial contamination is important e.g. in the food industry. Increased use of copper may lead to an increase in copper resistance in bacteria. However, copper and copper alloys have been used for many years without widespread increases in the reported incidence of copper resistant pathogens.

## Competing interests

CVD Technologies Ltd develop and install CVD coating equipment. The authors declare that they have no other competing interests.
